# A 3D Finite-Difference BiCG Iterative Solver with the Fourier-Jacobi Preconditioner for the Anisotropic EIT/EEG Forward Problem

**DOI:** 10.1155/2014/426902

**Published:** 2014-01-12

**Authors:** Sergei Turovets, Vasily Volkov, Aleksej Zherdetsky, Alena Prakonina, Allen D. Malony

**Affiliations:** ^1^Electrical Geodesics, Inc., Eugene, OR 97403, USA; ^2^Department of Computer and Information Science, 1202 University of Oregon, Eugene, OR 97403, USA; ^3^Department of Mathematics and Mechanics, Belarusian State University, 220050 Minsk, Belarus

## Abstract

The Electrical Impedance Tomography (EIT) and electroencephalography (EEG) forward problems in anisotropic inhomogeneous media like the human head belongs to the class of the three-dimensional boundary value problems for elliptic equations with mixed derivatives. We introduce and explore the performance of several new promising numerical techniques, which seem to be more suitable for solving these problems. The proposed numerical schemes combine the fictitious domain approach together with the finite-difference method and the optimally preconditioned Conjugate Gradient- (CG-) type iterative method for treatment of the discrete model. The numerical scheme includes the standard operations of summation and multiplication of sparse matrices and vector, as well as FFT, making it easy to implement and eligible for the effective parallel implementation. Some typical use cases for the EIT/EEG problems are considered demonstrating high efficiency of the proposed numerical technique.

## 1. Introduction

The progress in the forward and inverse modeling in Electrical Encephalography (EEG) and Magnetoencephalography (MEG) source localization as well as in Electrical Impedance Tomography (EIT) depends on efficiency and accuracy of the employed forward solvers for the governing partial differential equations (PDE), in particular, the Poisson equations, describing the electrical potential distribution in highly heterogeneous and anisotropic human head tissues.

The modern forward solvers use the variety of computational approaches based on the finite difference (FD), boundary element (BE), and finite element (FE) methods [1–9], multigrid [10] and preconditioned Conjugate Gradient- (CG-) type iterative methods [11–15], and also high performance parallel computing techniques [16–22].

To describe the electrical conductivity in heterogeneous biological media with arbitrary geometry, the method of embedded boundaries or a fictitious domain can be used [23, 24]. In this method, an arbitrarily shaped object of interest is embedded into a rectangular computational domain with extremely low conductivity values in the external complimentary regions modeling the surrounding air. This effectively guarantees that there are no current flows out of the physical area and implicitly sets up the zero flux Neumann boundary condition on the surface of the object. This setup retains the advantages of the finite-difference method (FDM), which is most prominent in the rectangular domain.

Previously, we built an iterative finite-difference forward problem solver for an isotropic version of the Poisson equation for EEG/EIT based on the vector-additive alternating directions implicit (ADI) algorithm [19]. It is a generalization of the classic ADI algorithm but with improved stability in the 3D case [25, 26]. Parallelization of the vector-additive ADI algorithm in a shared memory multiprocessor environment (OpenMP) is straightforward, as it consists of nests of independent loops over “bars” of voxels for solving the effective 1D problem at every iteration [19]. However, the ADI method is less suitable for implementation in an environment with a distributed memory. Therefore we also presented in the past an anisotropic vector-additive algorithm of the domain decomposition type [20] which is potentially amenable for implementation at the greater parallel degree [21].

The methods belonging to the family of the Conjugate Gradient (CG) methods [11–16] have become recently the most attractive iterative numerical techniques for solving the forward EEG/EIT problem. These methods have the high convergence rate to reach the required accuracy: the iteration number is proportional to the square root from the condition number of the system matrix in Linear Algebraic Equations (LAE) to solve. In case of a finite-difference discretization, the condition number of a system matrix is inversely proportional to the grid step squared resulting in an increase of the iteration number with an increase in the grid resolution. Additionally, the condition number depends on heterogeneity of coefficients in PDE, in particular, the ratio of maximal and minimal conductivities in the media. To reduce the condition number, one needs to employ the preconditioned CG-type iterative methods such as BiCG [13, 14] in the cases of strongly heterogeneous and anisotropic conductive media. In this paper we demonstrate an efficient way of preconditioning in FDM by using benefits of the Fast Fourier Transform (FFT) [11] technique as a tool for building a quasioptimal preconditioner for the CG-type iterative solvers. As a quasioptimal preconditioner, we suggest to use the spectrally adapted matrix of the corresponding Dirichlet problem with homogeneous isotropic coefficients in the same computational domain. Although this idea is not completely new (see, e.g., [23, 24]), to the best of our knowledge, it has been used so far in the EIT/EEG context only in our previous work on the isotropic cylinder forward solver [19]. The most attractive advantage of such a preconditioner is the ability to eliminate dependence of the convergence rate of the iterative method on the grid size. Additionally, we apply also the standard Jacobi preconditioner, which improves performance of the solver in the case of strongly heterogeneous conductivity coefficients. It is worth to note, that the spectrally adapted quasioptimal preconditioner based on FFT has no analogies in FEM or in the case of FDM in irregular domains and/or FDM with a nonuniform grid.

## 2. Methods

### 2.1. Mathematical Statement of the Problem

The relevant frequency spectrum in EEG, MEG, and EIT of the human head is typically below 100 kHz, and most EEG/MEG studies deal with frequencies between 0.1 and 100 Hz. Therefore, the physics of EEG/MEG can be well described by the quasistatic approximation of the Maxwell equations and the Poisson equation [3]. The electrical forward problem can be stated as follows: given the positions, orientations, and magnitudes of dipole current sources, *f*(*x*, *y*, *z*) as well as geometry and electrical conductivity of the head volume (Ω), calculate the distribution of the electrical potential on the surface of the head (scalp) (Γ_*Ω*_). Mathematically, it means solving the inhomogeneous anisotropic Poisson equation [2]:

(1a) $$\begin{array}{c} \nabla \cdot \left(\sigma \nabla u\right)=f\left(x,y,z\right),\;\left(x,y,z\right)\in \Omega \end{array}$$

with no-flux Neumann boundary conditions on the scalp:

(1b) $$\begin{array}{c} \sigma \left(\nabla u\right)\cdot n,\;\left(x,y,z\right)\in \Gamma_{\Omega }. \end{array}$$

Here *σ* = *σ*_*ij*_(*x*, *y*, *z*) is an inhomogeneous symmetric tensor of the head tissues conductivity. Having computed potentials *u*(*x*, *y*, *z*) and current densities *J* = −*σ*(∇*u*), the magnetic field *B* can be found through the Biot-Savart law for the MEG forward problem. The similar nonstationary anisotropic diffusion equation is relevant also in the diffusion optical tomography forward problem modeling [1], spread of tumor in brain [27], and the white matter tractography studies using diffusion tensor MRI imaging [28].

For the validation purposes of the suggested numerical solver for (1a) and (1b) we have employed several models of volume conduction, including a smooth analytical solution, an anisotropic multishell spherical model where analytical solutions are available [29], and anatomically accurate MRI based models of the human head as described below.

### 2.2. Smooth Analytical Solution

A simple exact analytical solution of (1a) and (1b) can be constructed assuming that in a cubic computational domain with edge length 2*a* the solution has the form

(2) $$\begin{array}{c} u\left(x,y,z\right)=\left(x-a\right)\left(x+a\right)\left(y-a\right)\left(y+a\right)\left(z-a\right)\left(z+a\right). \end{array}$$

Apparently, such a solution satisfies the Dirichlet boundary conditions at the computational domain boundaries. If the analytical conductivity tensor components in (1a) and (1b) are chosen in the form of the smooth analytical functions of the spatial variables, for example,

(3) σxx=6(2+x2+y2+z2),σyy=5(2+x2+y2+z2),σzz=4(2+x2+y2+z2),σxz=σzx=σyz=σzy=σxy=σyx=sin((x−1)(x+1)+(y−1)(y+1)  +(z−1)(z+1)),

then the right-hand term, *f*(*x*, *y*, *z*), can be found by direct analytical differentiation of the probe solution function, *u*(*x*, *y*, *z*), and coefficients (3), according to (1a).

### 2.3. Multishell Spherical Model

A 4-shell spherical model with an anisotropic skull layer was used to test the solver in case of highly heterogeneous anisotropic media. The model shells represent the scalp, skull, cerebral spinal fluid (CSF), and brain. Following Ferree et al. [30], the external radii of shells were chosen to be 0.084 (m, scalp), 0.065 (m, skull), 0.05 (m, CSF), and 0.03 (m, brain), and conductivity values used in the spherical model were set to 0.44 (S/m, scalp), 0.018 (S/m, skull), 1.79 (S/m, CSF), and 0.250 (S/m, brain). Consistent with recent evidence the skull to brain conductivity ratio was set to 14 : 1 [31–33], in contrast with the 80 : 1 ratio traditionally assumed [34]. We have chosen the largest tangential to radial conductivity ratio 1 : 10 reported in the literature [35] to push the limits of stability and robustness of our forward anisotropic solver. To find the coefficients of the anisotropic conductivity tensor in the global Cartesian system of coordinates we used the rotational transformations applied to the local coordinate systems, where the conductivity tensor has the diagonal form [36]. The conductivity tensor in such a model of anisotropy is a fair approximation of the cranial plates conductivity in the human head [3]. The main purpose of the spherical model use, however, was to validate the numerical solver against the analytical results [29] and demonstrate efficiency of the proposed numerical approach in the highly heterogeneous anisotropic case; therefore the shell thicknesses were chosen to be larger to facilitate comparison of numerical performance with the coarse and fine finite-difference of grid resolution.

### 2.4. Realistic MRI/CT Based Model

The anatomically accurate model of soft head tissues for an adult subject was derived from T1-weighted MR and DT images of the head of a healthy Caucasian male of 42 years old recorded with a 3T Allegra scanner (Siemens Healthcare, Erlangen, Germany) and stored in the Oregon Normative database (Electrical Geodesics, Inc.). The bone structure for this subject was derived from a CT scan recorded with a GE CT scanner (General Electrics, Fairfield, USA). The acquisition matrix has size 256 × 256 × 256 with a voxel size of 1 mm × 1 mm × 1 mm in both the CT and T1 scans. DTI was performed only for the cranial head part with a voxel resolution of 2 mm × 2 mm × 2 mm. To construct the isotropic head geometry, the T1 MRI images have been automatically segmented into seven tissue types (brain gray matter, brain white matter, CSF, scalp, eyeballs, air, and skull), coregistered, and warped with CT images using segmentation and image-processing package, BrainK [37, 38]. We estimated diffusion tensors from the raw diffusion weighted images with a least square fitting procedure using the TEEM software package [39]. To account for the white matter anisotropy, scalar diffusivity maps from the DTI were calculated and then rigidly registered to the T1 brain image with a mutual information metric [40–43]. Finally, using the same affine transformations the whole diffusion tensor field was resampled and aligned to the T1 image and its segmented tissue mask by utilizing log-Euclidean tensor interpolation [44]. Conductivity values used in this realistic model for isotropic tissues were set to 0.44 (S/m, scalp layer including eyes), 0.018 (S/m, skull), 1.79 (S/m, CSF), and 0.250 (S/m, brain). The conductivity tensor for the brain white matter is obtained directly as the product of white matter isotropic conductivity (*σ* ^iso^ = 0.25 S/m) and the coregistered diffusion tensor *D*_*w*_ scaled by its mean diffusivity (the tensor trace divided by 3) [5–8]:

(4) $$\begin{array}{c} \sigma_{\text{WM}}=3\cdot \sigma^{\text{iso}}\cdot \frac{D_{w}}{\mathrm{tr}\left(D_{w}\right)}. \end{array}$$

In this approach, the resulting white matter conductivity is both anisotropic and inhomogeneous due to the spatial dependence of the diffusion tensor eigenvalues.

### 2.5. Finite-Difference Method

**Figure 1:**
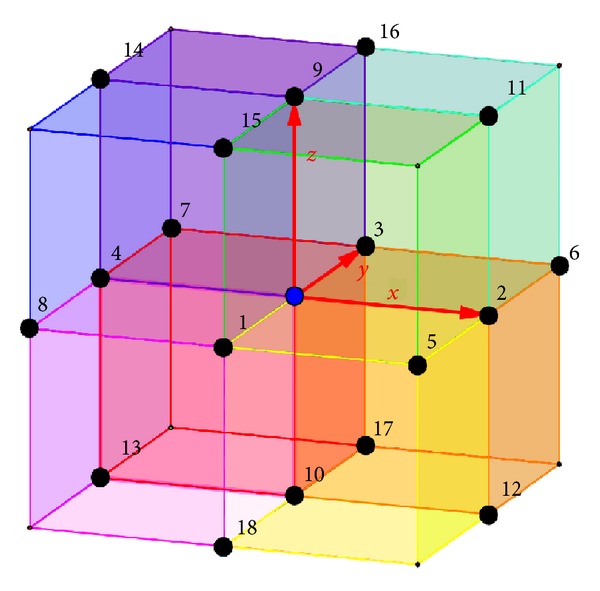
The finite-difference stencil of a discrete approximation for the anisotropic problem ((1a) and (1b)).

We have used finite-difference approximations of the spatial derivatives on the uniform rectangular grid with a 19-point stencil made of 8 voxels with one common node, as shown in Figure 1. All stencil nodes belong to three mutually orthogonal planes. Let us illustrate the discretization on the example of plane *Oxy*. To approximate the second derivatives we have used the standard conservative scheme for the finite volumes [25, 45]:

(5) ∂∂xσxx∂U∂x=hx−2[σxx02U2−(σxx02+σxx04)U0+σxx04U4]+O(hx2),

where *σ*_*xx*_^*km*^ = (*σ*_*xx*_^*m*^ + *σ*_*xx*_^*k*^)/2 and indices (superscripts and subscripts) refer to conductivity parameters and potentials in corresponding stencil nodes, as shown in Figure 1. To approximate the mixed derivatives we have investigated five kinds of the second-order accuracy schemes. As an example we present here these approximations only for one of the mixed derivatives:

(6a) ∂∂xσxy∂U∂y  =14hxhy  ×(σxy2(U6−U2)−σxy0(U3−U0)    +σxy0(U0−U1)−σxy4(U4−U8)    +σxy2(U2−U5)−σxy0(U0−U1)    +σxy0(U3−U0)−σxy4(U7−U4))  +O(hx2+hy2),

(6b) ∂∂xσxy∂U∂y =14hxhy  ×(σxy+2(U6−U2)−σxy+0(U3−U0)    +σxy+0(U0−U1)−σxy+4(U4−U8)    +σxy2(U2−U5)−σxy−0(U0−U1)    + σxy−0(U3−U0)−σxy−4(U7−U4))  +O(hx2+hy2),

(6c) ∂∂xσxy∂U∂y =14hxhy  ×(σxy26(U6−U2)−σxy03(U3−U0)    +σxy01(U0−U1)−σxy48(U4−U8)    +σxy25(U2−U5)−σxy01(U0−U1)    + σxy03(U3−U0)−σxy47(U7−U4))  +O(hx2+hy2),

(6d) ∂∂xσxy∂U∂y =14hxhy  ×(σxy2(U6−U5)−σxy4(U7−U8))  +O(hx2+hy2),

(6e) ∂∂xσxy∂U∂y =14hxhy  ×(σxy02(U6−U5+U3−U1)    − σxy04(U3−U1+U7−U8))  +O(hx2+hy2),

where *σ*_*xy*_^±*k*^ = *σ*_*xy*_^*k*^ ± |*σ*_*xy*_^*k*^|. One can see that in the homogeneous case of constant conductivity all of these approximations (except for case (6b)) are equivalent. In the case of inhomogeneous anisotropic media approximation (6a) is usually preferable due to its conservative nature [25], similar to the finite volume approximation used in (2). Finite-difference approximation (6b) is also conservative and satisfies the discrete maximum principle under some conditions [25, 46]. Finite-difference approximation (6c) is a simple modification of scheme (6a) with some additional grid points in the stencil (see Figure 1) for averaging the coefficients similar to the approximation in (2). Scheme (6d) is a generalization for the inhomogeneous case of the typical four-point approximation of mixed derivatives with constant coefficients. Finally, finite-difference approximation (6e) is a conservative scheme with an additional important property in comparison with ((6a)–(6d)): it uses the same stencil nodes for diagonal and off-diagonal conductivity tensor components. This property makes approximation (6e) more stable and ensures the positive definiteness of the resulting tensor approximation on the local scale for piecewise inhomogeneous anisotropic media which are typical for the multishell EEG/MEG/EIT forward models.

**Figure 2:**
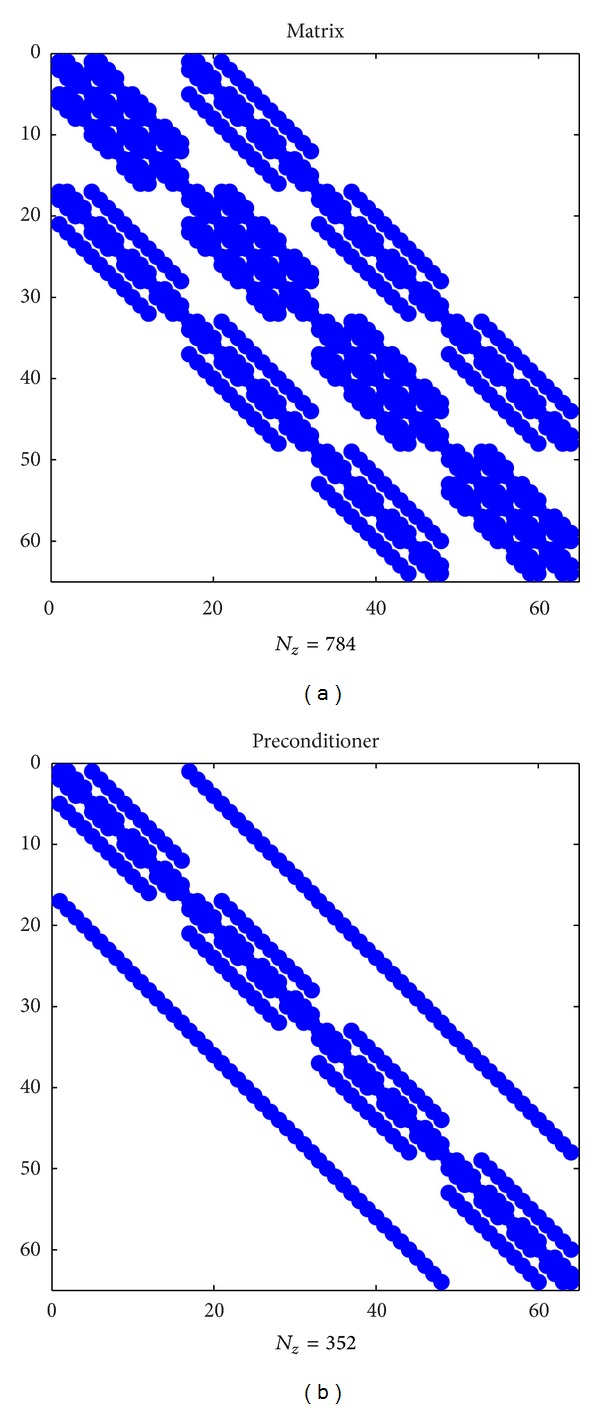
Structure of the system matrix (a) and the preconditioner (b).

For all cases under consideration, the discretized problem leads to solving a large system of LAE with the square 19-diagonal matrix (Figure 2(a)) of dimension *N* = *N*_*x*_ *N*_*y*_ *N*_*z*_, and iterative methods are the best option of choice to deal with such systems of LAE:

(7) $$\begin{array}{c} AU=f. \end{array}$$

The effectiveness of iterative methods is defined by the convergence rate (a number of iterations to achieve a given accuracy) as well as by the computational complexity of one iteration. The rate of convergence of the applied iterative methods depends on the condition number of the corresponding system matrix [12]. In the best case of the basic iterative methods the functional dependence of the convergence rate is the square root of the condition number for the system matrix *A* of the problem. Such a high convergence rate, for instance, is inherent to the CG iterative methods in the case of the Hermitian system matrix. If the system matrix is nonsymmetric, the modifications of the CG methods such as BiCG or BiCGStab can be used [14].

For systems of LAE arising in the finite-difference numerical approximations for PDE, the condition number is inversely proportional to the grid step squared [12, 25]. In addition, heterogeneity of coefficients in PDE leads to a further increase in the condition number and makes the system matrix nonsymmetric. Due to these reasons, the preconditioned BiCG iterative method is an option of choice in PDEs with highly heterogeneous coefficients on the high resolution grid. Generally, the preconditioner is a nonsingular matrix *P*, such that the condition number of the matrix *B* = *P* ^−1^ *A* is much smaller than the condition number of the original matrix *A*. The use of preconditioners is in fact equivalent to the transition from the original problem (7) to the following problem:

(8) $$\begin{array}{c} P^{-1}AU=BU=P^{-1}f. \end{array}$$

Another important criterion for selecting a good preconditioner (in addition to its primary function of reducing the condition number) is a computational cost of inverse matrix calculation. In this respect, preconditioners in the form of the diagonal, triangular, or sparse circulant matrices are most attractive. In the latter case the fast inverse matrix calculation can be achieved by use of the fast discrete Fourier transform.

As one of the simplest forms of preconditioning almost not requiring additional computations one can suggest the Jacobi-type (diagonal) preconditioner [13, 14]. It allows reducing a number of iterations when solving PDEs with strongly inhomogeneous coefficients like in the Dirichlet problem in the fictitious domain we are dealing here. However, the Jacobi preconditioner does not damp the increase in a number of iterations with an increase of the grid resolution. In addition to the Jacobi preconditioner, one can use as a preconditioner the system matrix corresponding to the case of the homogeneous isotropic limit. This matrix (also known as the 3D Poisson matrix [12, 25]) has the 7-diagonal circulant form (see Figure 2(b)). Because the Fast Fourier Transform (FFT) can be employed to compute an inverse matrix of such a preconditioner, it is referred to as the *Fourier preconditioner* [23, 25]. Along with image reconstruction problems, the Fourier preconditioner is successfully used in numerical analysis of PDEs [13, 23] including the Poisson equation in EIT/EEG [16]. In many cases, the Fourier preconditioner allows eliminating dependence of an iteration number to convergence from the grid resolution [16, 23], similar to the case of the multigrid preconditioner [10]. We have checked efficiency of the above-mentioned types of preconditioned BiCG iterative methods using their standard realization in MATLAB [16, 17]. Taking into account the uniform Dirichlet boundary conditions we have modified the module of postprocessing for the Fourier preconditioner by use of the sin-Fourier transform, which allows presenting the preconditioner matrix in the diagonal form.

## 3. Results: Validation and Numerical Examples

**Figure 3:**
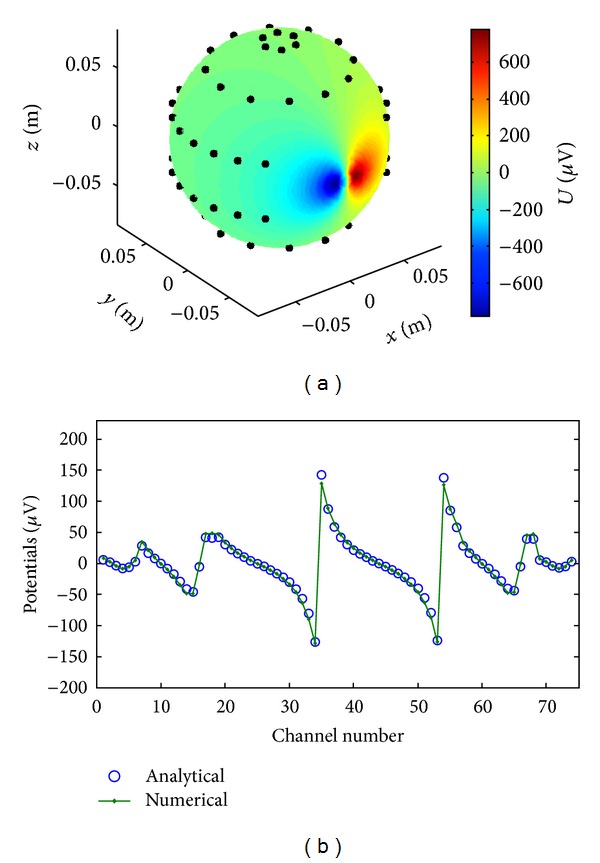
Validation of the numerical scheme against the analytical solution in the anisotropic spherical model [29]. The FD computed potentials (solid) and analytical (open circles) curves versus channel number.

Numerical modeling of smooth analytical probe solutions ((2) and (3)) for the PDE problem ((1a) and (1b)) has proved to be of the second order of approximation in regard to the grid step for all five approximations of mixed derivatives ((6a)–(6e)). We have also validated the numerical methods against the analytics in the layered anisotropic spherical model [29]. Based on these simulation tests we have derived the performance figures for the suggested numerical method. The anisotropic spherical head was embedded into the fictitious cubic computational domain with the edge length of 0.1 m padded with dielectric media (air) with the conductivity of 10^−10^ S/m. Sensors were distributed evenly along the geodesic lines. The resulted computed scalp topography is shown in Figure 3(a) for a source—sink pair placed on the equator in the middle of the outer shell. In Figure 3(b) the simulated topography is compared with an approximate analytical solution [29]. The results show a good agreement of the numerical solution with the analytics with some perceptible deviations near the dipole source, where it might be expected due to difference in discrete and analytical current source approximations.

**Figure 4:**
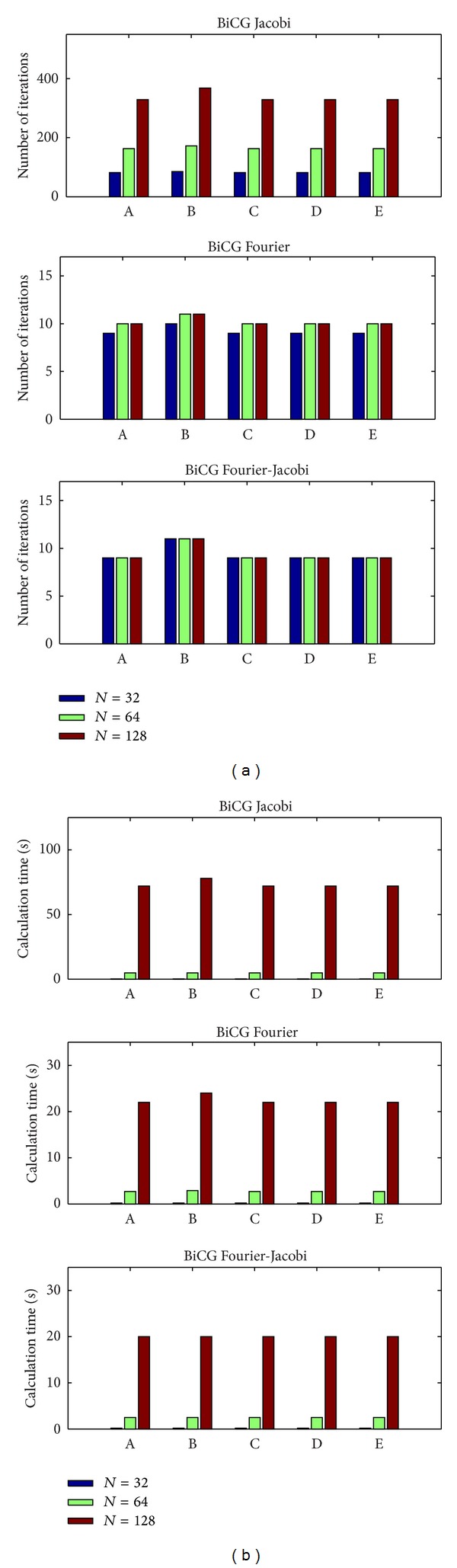
Efficiency of different preconditioners for inhomogeneous smooth solutions (an analytical probe function (2)). Number of iterations (a) and runtime in seconds (b) versus a mixed derivative approximation type ((6a)–(6e)) for different resolutions (*N* ^3^).

**Figure 5:**
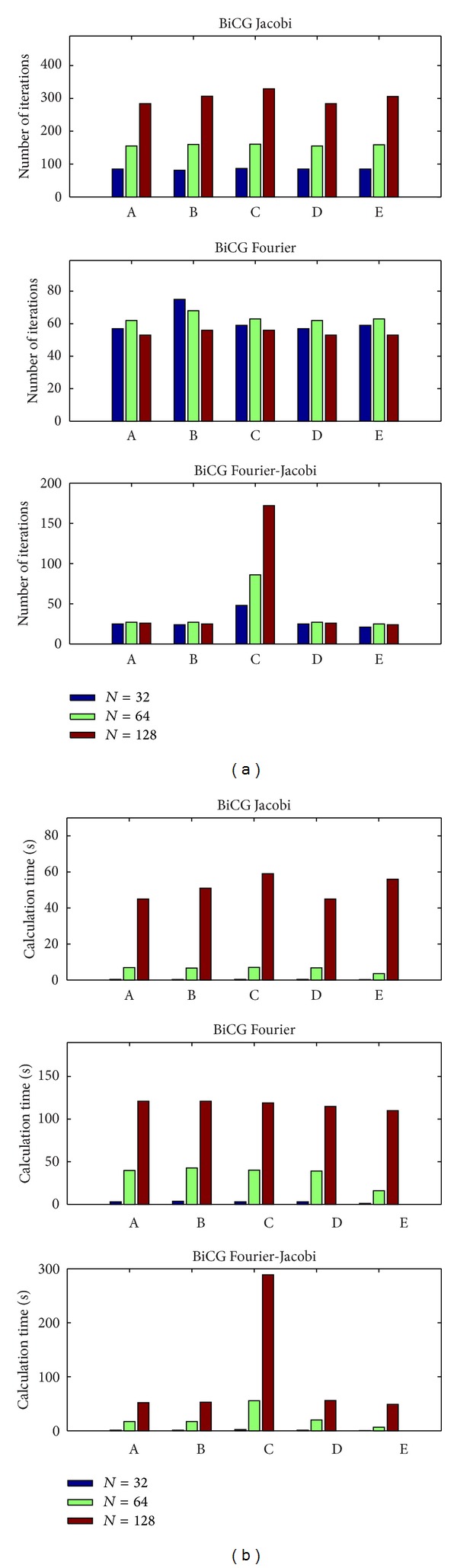
Efficiency of different preconditioners for the piecewise heterogeneous anisotropic spherical model (see Figure 3). Number of iterations (a) and runtime in seconds (b) versus a mixed derivative approximation type ((6a)–(6e)) for different resolutions (*N* ^3^). Approximation C is outperformed by others.

To study the performance of the suggested method we have investigated dependence of the convergence rate on the grid resolution and the type of the finite-difference approximation. The number of iterations and the total computation time required for achieving the given accuracy as a function of a grid node number are shown in Figures 4 and 5.

For the smooth Dirichlet-type solutions the BiCG method with the Fourier and Fourier-Jacobi (FJ) preconditioners requires 9–11 iterations independently of the grid resolution and outperforms by a large factor the BiCG method with and without the Jacobi preconditioner in terms of the computational time. The computational time per iteration in the case of the Fourier preconditioners is of one order of magnitude larger than in the BiCG method without or with the Jacobi preconditioner. However, when using the Jacobi preconditioner the number of iterations to convergence increases proportionally to the number of grid nodes. For *N* = 128 the number of iterations in the Jacobi case exceeds 300 and the total computational time to solve the problem is about 3 times larger than the time required for the Fourier preconditioner case (Figure 4). The number of iterations to convergence is approximately the same for all five mixed derivatives approximations ((6a)–(6e)) in all three preconditioned BiCG methods.

When the piecewise heterogeneous 4-shell spherical model is used, the performance depends on the choice of a discrete approximation for the mixed derivatives ((6a)–(6e)). The more stable and robust results were obtained with the approximation of type (6e), where the FJ preconditioner effectively reduces the number of iterations to convergence and removes the dependence from the grid resolution. For instance, the iteration number required to reach the accuracy of *ε* = 10^−5^ is not larger than 30 and does not depend on the grid resolution. At the same time with approximation (6c) the FJ preconditioner loses its scaling property and that reflects in dependence of iteration number from the grid resolution. It is worth to note that the Jacobi preconditioners for this problem also demonstrate the good performance at the relatively coarse resolution of the discrete model. For example, in the case of 128 points in each spatial direction, the total computational cost per iteration when using the Jacobi and Fourier-Jacobi preconditioners is approximately the same, but, due to the iteration number dependence on the resolution in the BiCG-J method, the BiCG-FJ method is more preferable for the higher grid resolutions. Also, the convergence of the FJ-BiCG methods has a very weak dependence on the anisotropy ratio. The average iteration number to convergence in case of the 4-shell spherical model with an anisotropic skull layer increases from 20 at the skull anisotropy ratio 1 : 1 to 25 at 1 : 10 and 30 at 1 : 50.

**Figure 6:**
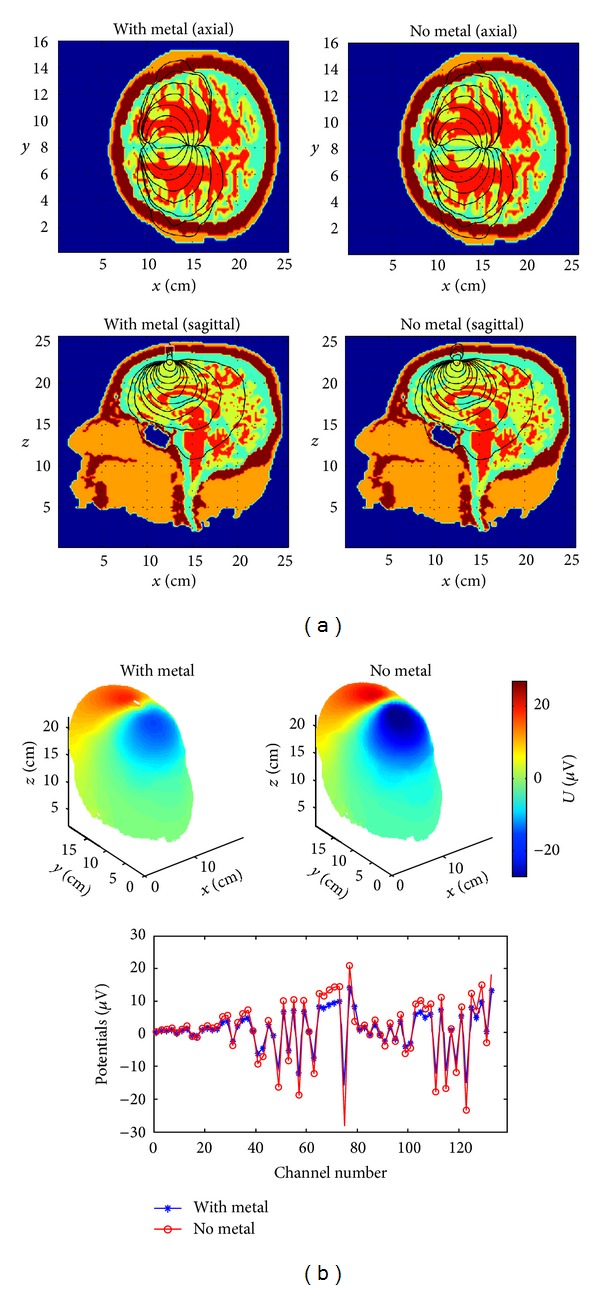
(a) Modeling of impact of the titanium surgical clip (the white “pi”) on the EEG forward solution in a “virtual” postoperational patient. A horizontally oriented dipole is of 0.6 cm from the clip. The shunting effect is getting more pronounced with the closer distance between the dipole and the clip and parallel orientation as it can be seen through the distortion of current stream lines (in black). (b) Impact of titanium surgical clip (the white “pi”) on the EEG forward solution in a “virtual” post-operational patient. 3D topography view (top) and 1D voltages (*μ*V) versus channel number detailed quantitative comparison (bottom). A horizontally oriented dipole is of 0.6 cm from the clip. The shunting effect is getting more pronounced with the closer distance between the dipole and the clip and parallel orientation as it can be seen through the distortion of the equipotential lines on scalp.

**Figure 7:**
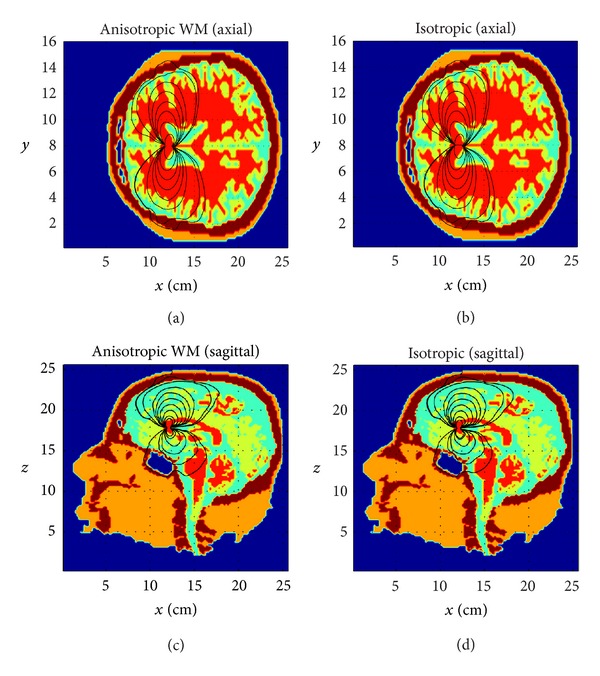
Impact of brain white matter (the red tissue) anisotropy on the EEG forward solution: axial views (top); sagittal views (bottom). The white matter is anisotropic (left) and isotropic (right). A horizontally oriented dipole is placed deep in the central brain region. The distortion of current stream lines can be seen in the anisotropic case (left) relative to the isotropic case (right).

**Figure 8:**
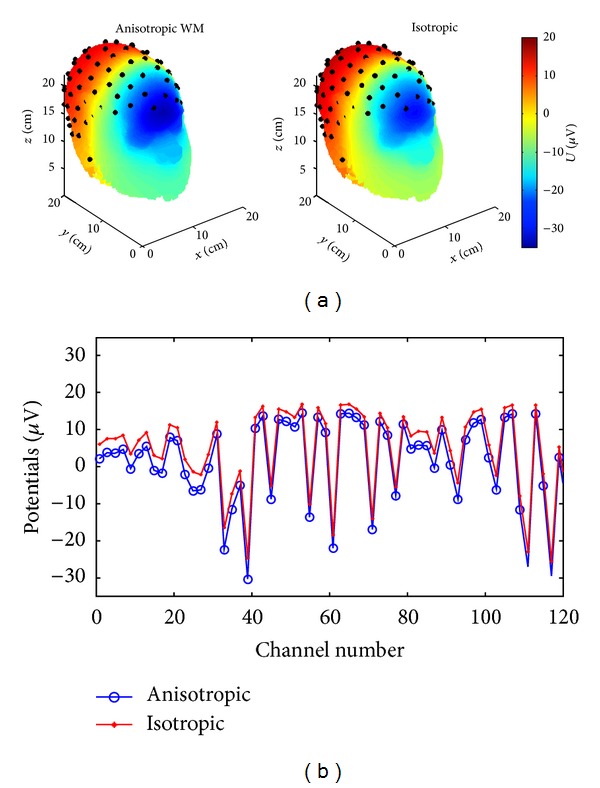
Impact of brain white matter anisotropy on the EEG forward solution: 3D topography view (a) and 1D voltages (*μ*V) versus channel number detailed quantitative comparison (b). A horizontally oriented dipole is placed deep in the central brain region.

We have also tested our solver in the realistic MRI/CT based human head multishell models derived from the high resolution (1 mm^3^) raw and segmented MRI volumes coregistered with CT atlases as described in Section 2. Sensors on the scalp were distributed evenly along the geodesic lines.First, simulation was done with a virtual metal surgical clip in skull (shaped as the Greek letter “Π” with the dimensions 12 mm × 12 mm × 12 mm, the cross-section of 2 mm × 4 mm, and titanium conductivity 2.5e6 S/m,) to test the limits of the solver tolerance to heterogeneity. As it is shown in Figure 6(a), the introduction of highly conductive metal clips into a head model leads only to modest local distortions of the EEG topography, similar to some extent to the impact on EEG of surgical burrs in skull [9]. The shunting effect is getting more pronounced with the closer distance between the dipole and the clip and their parallel orientation as it can be seen through the distortion of the equipotential lines on scalp in Figure 6(b). It is noteworthy that the modeling of the EIT/EEG problems in presence of metal implants using the fictitious domain with air claddings results in the extremely high heterogeneity ratio of the order of ~10^10^−10^16^ and the ill-posed system matrix of the discrete model. Our previous FDM version of the forward EEG/EIT solver based on the ADI method [19] diverged at such high levels of heterogeneity. However, the use of the BiCG-FJ method and the robust mixed derivatives approximations of type (6a) and (6e) allows to reach an approximate solution after 60–80 iterations, while the other preconditioners get quickly outperformed with an increase in the grid resolution. Second, as a test case, we have modeled an impact of the brain white matter anisotropy and inhomogeneity on EEG using the brain white matter conductivity tensor inferred from DTI in accordance with (4). As can be seen in Figures 7 and 8, the resulting current streamlines and scalp topographies are visibly different. The more quantitative analysis of our deep brain dipole simulation presented in Figure 8(b) shows that the isotropic brain white matter model versus the anisotropic one introduces an error of up to 25% for the lead fields on scalp in a qualitative agreement with the earlier published results on modeling the white matter anisotropy [4–6]; therefore anisotropic lead field calculations are important to introduce to increase the source localization accuracy in functional neuroimaging such as EEG or MEG [6]. It is worth noting here that reports on quantitatively different effects of white matter anisotropy depending on the model employed continue to appear in the literature, in particular in publications on transcranial electrical stimulation [47, 48] and EEG [7]. Lee et al. [7] reported a quite moderated impact of the white matter anisotropy on the source localization accuracy confirmed by fMRI. Thus, the further refinement of models and algorithms capable of dealing accurately with anisotropy is still in the process of ongoing improvement and constitutes a significant goal.

## 4. Discussion and Conclusion

In context of modeling EEG/EIT problems there are several most discussed and usable in practice numerical methods. These are the sequential overrelaxation (SOR), the CG-type methods such as Bi-Conjugate Gradient and Bi-Conjugate Gradients Stabilized methods with reasonable preconditioners (Symmetric Successive Overrelaxation (SSOR), Incomplete Cholesky and Incomplete Lower Upper (IC/ILU), factorization, Jacobi, Block Jacobi, and so on) and the algebraic multigrid (AMG) iterative methods. The efficiency of these approaches for the cases of isotropic and anisotropic problems is reviewed in [15, 22], where it is shown that the best performance is demonstrated by the AMG methods. The key advantage of the AMG methods is independent of the convergence rate on the computational grid resolution.

In this paper we have presented a novel type of an EEG/EIT anisotropic FDM forward solver from the CG methods family. The combined Fourier-Jacobi preconditioner shows unprecedented performance and robustness comparable with the AMG methods when applied to the problems with high heterogeneity and anisotropy. It is capable of solving 128 × 128 × 128 voxels anisotropic problems with the extreme conductivity tensor eigenvalues ratio of 10 : 1 and the isotropic tissue heterogeneity ratio of up to 10^16^ (including explicitly titanium clips and air pockets modeling) within a minute runtime in the MATLAB implementation. The high performance of the proposed method is due to the spectral equivalence property of the Fourier-Jacobi preconditioner. Its combination with the BiCG method eliminates the dependence of the convergence rate on the spatial grid resolution and the heterogeneity ratio of the discrete model. The number of iterations to achieve the desired accuracy is almost independent of the grid resolution, which puts the proposed technique in line with the popular multigrid iterative methods [3, 10, 15, 22]. The proposed numerical algorithm includes the standard operations of summation and multiplication of sparse matrices and vectors as well as FFT, making it easy to implement and readily eligible for the effective parallel implementation.

It is shown that certain types of difference approximations of the anisotropic problem in cases with strong heterogeneity of the coefficients of the problem do not support the optimal spectral property of the Fourier-Jacobi preconditioner. We believe that it is associated with the heritability in a discrete model of the fundamental properties of symmetry and positive definiteness of the conductivity tensor. When this property is lost in a particular kind of a discrete approximation ((6a)–(6e)), for instance, in approximation (6c), it adversely affects the matrix property in the discrete model and the efficiency of iterative methods for solving the problem. This is supported by the fact that the most reliable results are obtained with the difference approximations, where the diagonal and off-diagonal components of the conductivity tensor are averaged over the same stencil points of the finite-difference grid (approximation (6e)). For a realistic MRI based model in the EEG and EIT applications, our simulation results show that the introduction of the white matter anisotropic conductivity derived from a spatially inhomogeneous diffusion tensor can change isotropic lead fields on scalp, up to 25%, while the highly conductive metal surgical clips perturb EEG potentials mostly locally. The suggested solver can also find applications in the field of transcranial electrical stimulation [47, 48], where accurate modeling is important to predict current densities delivered to regions of interest on cortex.
